# Highly Performance Core-Shell TiO_2_(B)/anatase Homojunction Nanobelts with Active Cobalt phosphide Cocatalyst for Hydrogen Production

**DOI:** 10.1038/s41598-017-15134-w

**Published:** 2017-11-06

**Authors:** Guang Yang, Hao Ding, Jiejie Feng, Qiang Hao, Sijia Sun, Weihua Ao, Daimei Chen

**Affiliations:** Beijing Key Laboratory of Materials Utilization of Nonmetallic Minerals and Solid Wastes, National Laboratory of Mineral Materials, School of Materials Science and Technology, China University of Geosciences, Xueyuan Road, Haidian District, Beijing, 100083 P.R. China

## Abstract

In this paper, a highly efficient core-shell structure of TiO_2_(B)/anatase photocatalyst with CoP cocatalyst has been synthesized via hydrothermal processes and a mechanical milling method. The designed core-shell TiO_2_(B)/anatase photocatalysts exhibit excellent performance by compared with pure TiO_2_(B) and anatase phase. With the participation of CoP particles, there is drastically enhanced  photocatalytic activity of TiO_2_(B)/anatase, and the H_2_-production rate can be up to 7400 μmol·g^−1^, which is about 3.2 times higher than TiO_2_(B)/anatase photocatalyst. The improved activity is attributed to the contribution of the well-matched core-shell structure and cooperative effect of CoP cocatalyst. The photogenerated holes of anatase can migrate more promptly to the adjacent TiO_2_(B) core than the photogenerated electrons, which result in an accumulation of electrons in the anatase, and CoP nanoparticles can contribute significantly to transferring electrons from the surface of TiO_2_(A). It was found that the efficient separation of electron-hole pairs greatly improved the photocatalytic hydrogen evolution in water under UV light irradiation.

## Introduction

Photocatalytic hydrogen production from water using solar energy has received increasing attention because this strategy is considered to be a globally accepted way for solving the energy problem^1–7^. Titanium dioxide (TiO_2_), especially anatase phase photocatalysts, has attracted significant attention in photocatalytic hydrogen evolution due to its low cost, excellent chemical stability and superior photocatalytic activity^8,9^. Nevertheless, TiO_2_ can only be motivated by UV-light due to its large band gap (3.2 eV), and the fast recombination of photogenerated electrons and holes limits the application of TiO_2_. Therefore, many researchers focus on TiO_2_ to make it sensitive to visible light and to inhibit the recombination of photogenerated charge carrier, which affect its overall photocatalytic efficiency.

Construction of homojunction photocatalysts might be a common and effective way to improve the separation efficiency of photoinduced electron-hole pairs. The commercially available photocatalysts, such as Degussa P25 TiO_2_, has been found to exhibit a better photoactivity than pure anatase in many reaction systems. The efficient electron and holes transferring between the two phases can increase lifetime of electrons and holes^10–13^. Coincidentally, a relatively new TiO_2_(B) crystal phase is observed, which shows different crystal structures with anatase, rutile, and brookite. Although the photocatalytic efficiency of TiO_2_ (B) is lower than anatase, the former phase possesses a narrow bandgap and specific conduction band (CB) and valence band (VB) edge potential. Thus, the potential difference between the CB and VB edges of the two phases can promote charge transfer from one phase to the other when TiO_2_(B) and anatase combined with each other forming a homojunction^14^. Li *et al*. synthesized core-shell anatase/TiO_2_(B) nanofiber, which shows enhanced photocatalytic activity compared to either single-crystal anatase or single-crystal TiO_2_(B) nanofiber^15^. Li’s group synthesized TiO_2_(B)-anatase homojunction with disordered surface shell, and the excellent performance for photocatalytic hydrogen evolution was 580 μmol h^−1^ under simulated solar light irradiation (0.02 g photocatalyst)^16^.

Although homojunction can improve the charge transfer, the H_2_-production efficiency of TiO_2_(B)/anatase is still limited because the accumulated electrons in CB may recombine with the holes in VB easily. So, it is a wide and effective strategy to load proper oxidation or reduction cocatalysts on the surface of semiconductor photocatalysts^17–19^. Various kinds of cocatalysts have been applied to TiO_2_ photocatalysts to improve the activity of H_2_ evolution reactions, including metal cocatalysts, metal oxide/sulfide cocatalysts and noble metals (e.g. Pt, Ag, Au and Ru)^20–25^. However, the preparation of these cocatalysts particles is complicated and quite expensive for widespread practical application. A variety of non-precious cocatalysts have been reported, including MoS^26^, Cu(OH)^27^, NiS^28^, NiO^29^. Recently, transition metal phosphides, including Ni_2_P, CoP, FeP, MoP and Cu_3_P have been exploited in electrocatalysis of the hydrogen evolution reaction, and achieved outstanding efficiency^30–32^. During these pioneering works, it has been noticed that CoP nanoparticles show good electrical conductivity with metallic behavior. Chen’s group discovered that CoP nanoparticles together with CdS nanorods exhibited excellent efficiency for photocatalytic hydrogen evolution^33,34^. We envision that the hydrogen production performance can be dramatically improved by choosing CoP cocatalysts nanoparticles.

Herein, we designed TiO_2_(B)/anatase core-shell homojunction nanobelts using CoP nanoparticles as cocatalysts for efficient photocatalytic H_2_ production from aqueous methanol solution. The electrons migrate to the same destinations are times longer than holes in TiO_2_(B)/anatase system, and the accumulated electrons can be easily separate and transfer with the participation of CoP cocatalyst. The subsequently photocatalytic H_2_-production reduction reactions can be effectively enhanced. So, it is believed that this strategy is feasible and will have great practical application for hydrogen production.

## Experimental

### Synthesis of core-shell structure photocatalyst

Core-Shell TiO_2_ nanobelts were obtained using a previously reported procedure^35^. 3 g of anatase powder was mixed with 40 mL of 10 M NaOH solution. The suspension were dispersed in an ultrasonic bath for 30 min then transferred into into a Teflon-lined stainless steel autoclave, sealed and maintained at the temperature of 180 °C for 48 h. The precipitate (sodium titanate nanobelts) was collected, washed with distilled water several times to remove excess NaOH. Then, the obtained precursor was exchanged with H^+^ using a 0.1 M HCl solution for 24 h to form H_2_Ti_3_O_7_ nanofibers. The product washed again with distilled water to neutral and dried at 70 °C for 10 h. The core-shell photocatalyst was synthesized by hydrothermal treatment of precursors in an acid environment and further subjected to heating process. 0.8 g of H_2_Ti_3_O_7_ nanofibers were dispersed in a dilute (0.05 M, 80 mL) HNO_3_ acid solution and kept at 110 °C for 48 h. The dried powder was heated in a muffle oven at 450 °C (2 °C/min) in air for 4 h, named T (AB). Reference samples: (1) Anatase catalyst was prepared by prolonging the hydrothermal reaction (HNO_3_ acid solution) time to 60 h and calcining at 450 °C in air for 4 h, noted as T (A); (2) TiO_2_ (B) catalyst was obtained by using dried H_2_Ti_3_O_7_ powder heated at 450 °C for 4 h, noted as T (B).

### Synthesis of CoP nanoparticles

CoP nanoparticles were prepared via a thermal phosphidation reaction using Co(OH)_2_ as precursor^33^. 200 mg of Co(NO_3_)_2_·6H_2_O was added to 100 mL aqueous solution containing 50 mg sodium citrate and stirred for 15 min. Then excess NaOH solution (0.5 M) was added to the mixture dropwise. The formed Co (OH)_2_ suspension was separated by centrifugation and dried at vacuum oven. Afterwards, 50 mg of obtained Co (OH)_2_ and 250 mg NaH_2_PO_2_ solid were ground in a mortar to form a uniform distribution and put in a quartz boat. Subsequently, the samples were maintained in tube furnace at 300 °C for 1 h with a heating rate of 2 °C·min^−1^ in a flowing Ar atmosphere (30 mL/min). Following cooling to room temperature, the obtained black solid was washed subsequently by water and ethanol three times and dried at vacuum oven.

### Loading of CoP Cocatalyst

**Figure 1:**
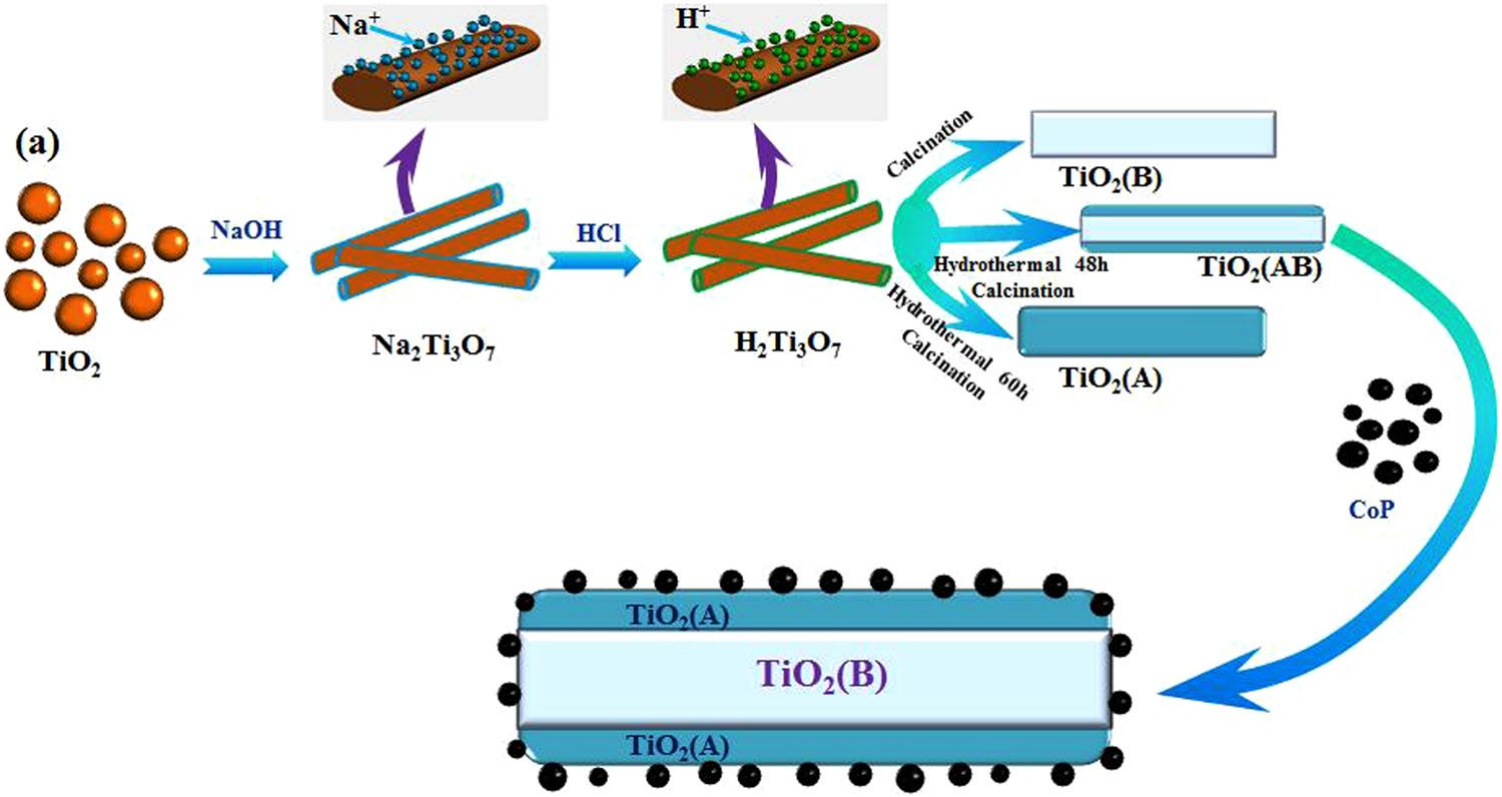
The preparation process of core-shell structure CoP/TiO_2_(AB) photocatalyst.

The loading of CoP nanoparticles on TiO_2_(B)/anatase core-shell photocatalyst was conducted by a mechanical milling method, the preparation process as shown in Fig. 1.

### Characterizations

X-ray diffraction (XRD) datas of the as-synthesized samples were recorded by a Brukeer D8A A25X X-ray diffractometer system using Cu Kα (λ = 0.15406 nm) radiation. The morphologies of the prepared samples were performed using a Hitachi SU-8010 instrument. High-resolution transmission electron microscopy (HR-TEM) and energy-dispersive X-ray spectroscopy (EDS) were applied to investigate the microstructure using a Tecnai G^2^ F30 instrument (HR-TEM operated at 200 kV). The UV-vis diffuse reflectance (DR) spectra of the samples were tested on a Hitachi U-3010 double beam spectrophotometer. The photoluminescence (PL) spectra were measured on a high-resolution multi-function imaging spectrometer (iHR 550) using laser transmitter (532 nm). X-ray photoelectron spectroscopy (XPS) was performed on an K-Alpha spectrometer (THERMO FISHERSCIENTIFIC).

### Photocatalytic H_2_-production

The photocatalytic H_2_-production experiment was carried out as follows, 100 mg photocatalyst and 10 mL methanol were added into the tube. Then the total volume of the mixed solution was adjusted to 100 mL with distilled water. A 300 W Xe lamp was used as light source and the electric current recorded to be 15A. Before light irradiation, the gas-tight system kept for several hours to make sure the tightness of all system and adsorption-desorption equilibrium between the photocatalyst and gases. The gas products were periodically analyzed by using a gas chromatography (GC-2014, Shimadzu Corp, Japan), which was equipped with a 5 Å molecular sieve column (3 m × 2 mm).

**Figure 2:**
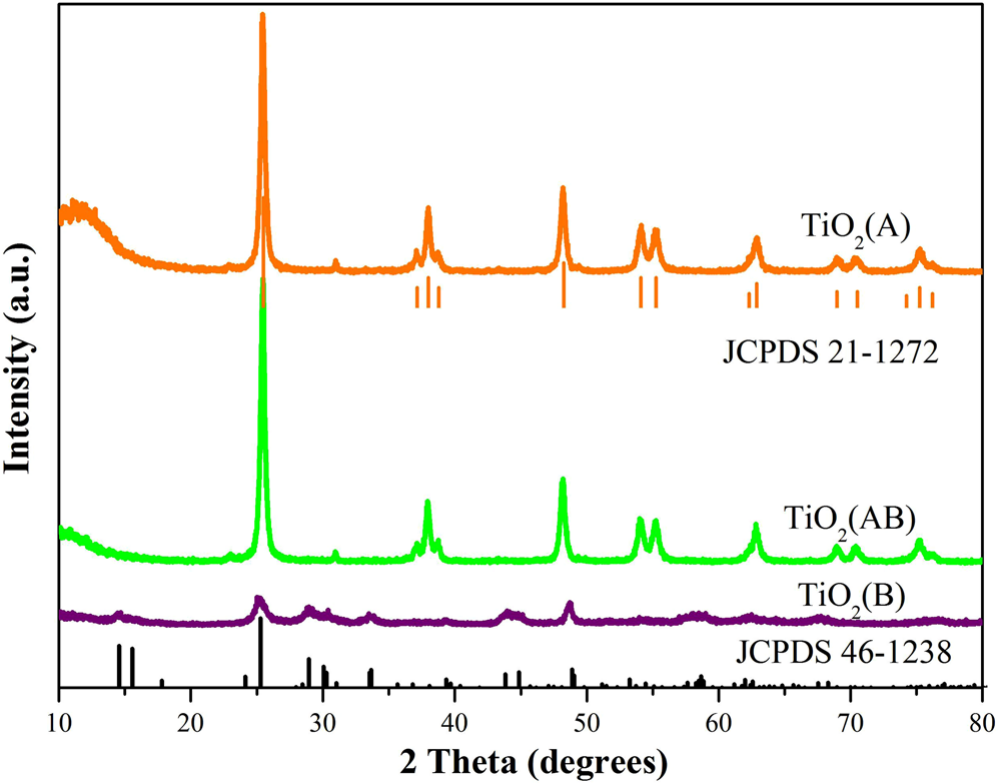
XRD patterns of TiO_2_(B), TiO_2_(A) and TiO_2_(AB) phase.

## Results and Discussion

Figure 2 shows the XRD patterns of the as-prepared TiO_2_(A), TiO_2_(AB) and TiO_2_(B) nanobelts. The obtained TiO_2_(A) and TiO_2_(B) samples are matched well with the crystal structure data of anatase phase (JCPDS No. 21–1272) and TiO_2_(B) phase (JCPDS No. 46–1238)^36,37^. The peak of TiO_2_(B) in the core-shell structure is not obvious may attribute to the overlapped main peak between TiO_2_(B) and TiO_2_(A), or the low contents of TiO_2_(B) for detection. In addition, as depicted in Figure S1, the XRD diffraction peaks of CoP nanoparticles are indistinguishable for each sample from CoP(0.5%)/TiO_2_ to CoP(5%)/TiO_2_ due to the low loading amount and weak crystallization of CoP nanoparticle. Similar phenomenon has been observed for other photocatalysts^33,38^.

The scanning electron microscopy (SEM) characterization can provide the direct evidence for the coexistence of CoP and TiO_2_. It can be seen that CoP nanoparticles are polydisperse (Figure S2a) and TiO_2_(AB) are nanobelts-like morphologies (Figure S2b). After coating with CoP nanoparticles, Co and P elements are distributed well on the surface of TiO_2_(AB) nanobelts photocatalyst (Figure S2c,d). The CoP-TiO_2_(AB) composites structure can be further confirmed in the EDS mappings analysis presented in Figure S2e. The results indicate that the Co, P, Ti and O elemental are uniformly distributed, and no other elements are detected. Furthermore, the corresponding energy dispersive X-ray spectroscopy (EDX) analysis confirms the existence of CoP and TiO_2_. However, the content of the measured CoP is lower than theoretical values in the final composites because of only surface CoP atoms can be checked (see Supplementary Figure S3).

**Figure 3:**
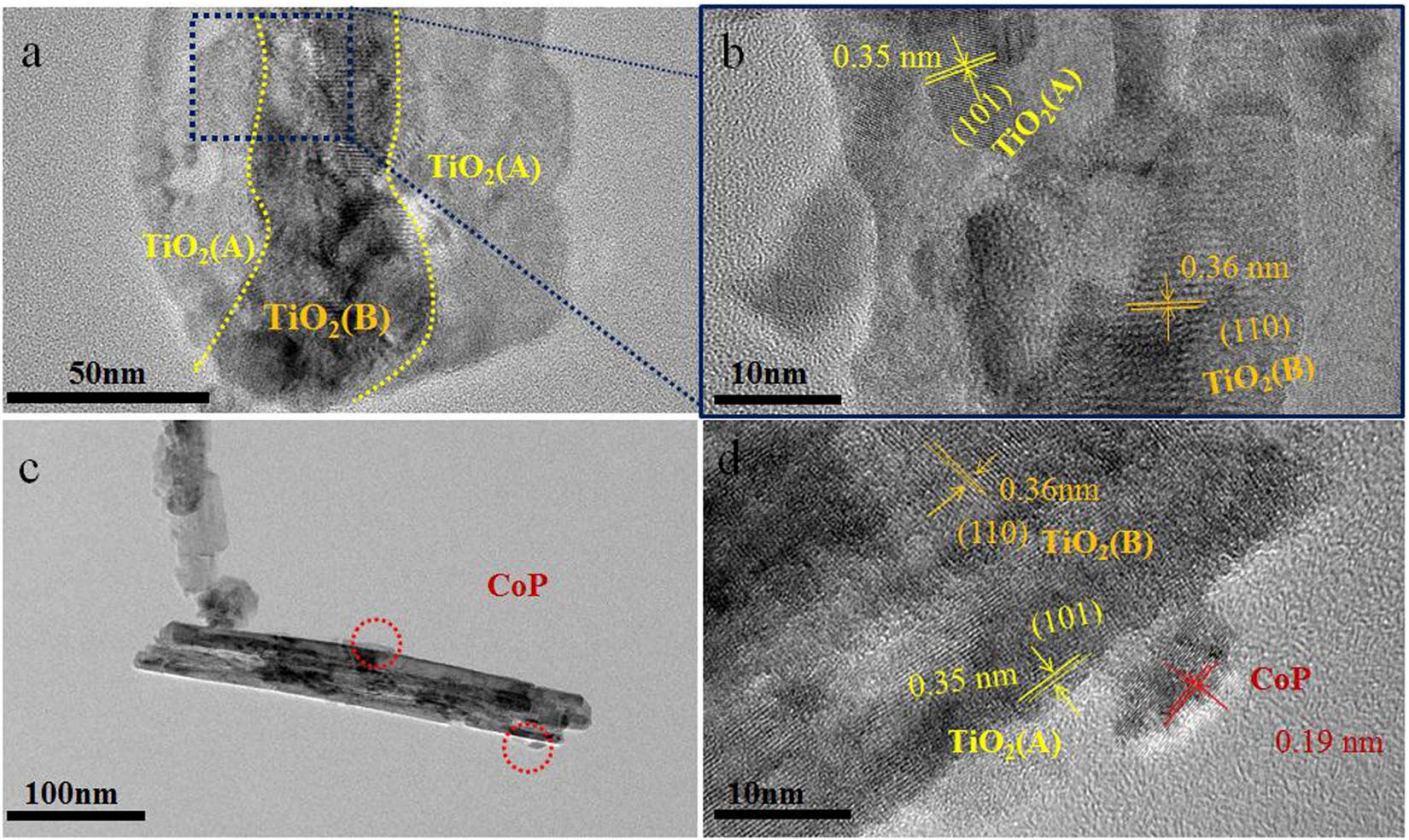
(**a**,**b**) TEM images of TiO_2_(AB) photocatalysts; (**c**,**d**) TEM and HRTEM image of CoP(1%)-TiO_2_(AB) photocatalysts.

To investigate the existence of core-shell homojunction, more explicit structure evolution is shown in transmission electron microscopy (TEM). As described in Fig. 3a, the typical TiO_2_(B)/anatase core-shell nanobelts can be observed clearly. The core of the nanobelts is corresponding to TiO_2_(B) phase, while the shell conforms to anatase phase. The HRTEM confirms that the sample possesses a homojunction structure with well-matched lattice fringes of the (110) plane of TiO_2_(B) and (101) plane of anatase (Fig. 3b). The TEM images of CoP(1%)-TiO_2_(AB) sample as depicted in Fig. 3c clearly indicate CoP nanoparticles (the red circle) are deposited on the surface of TiO_2_(AB) photocatalyst. The HRTEM image further shows that TiO_2_(AB) and CoP nanoparticles are in close contact with each other (Fig. 3d). The lattice spacings of ca. 0.35 nm and 0.19 nm belong to the diffraction planes of TiO_2_(A) and CoP nanoparticles, respectively.

**Figure 4:**
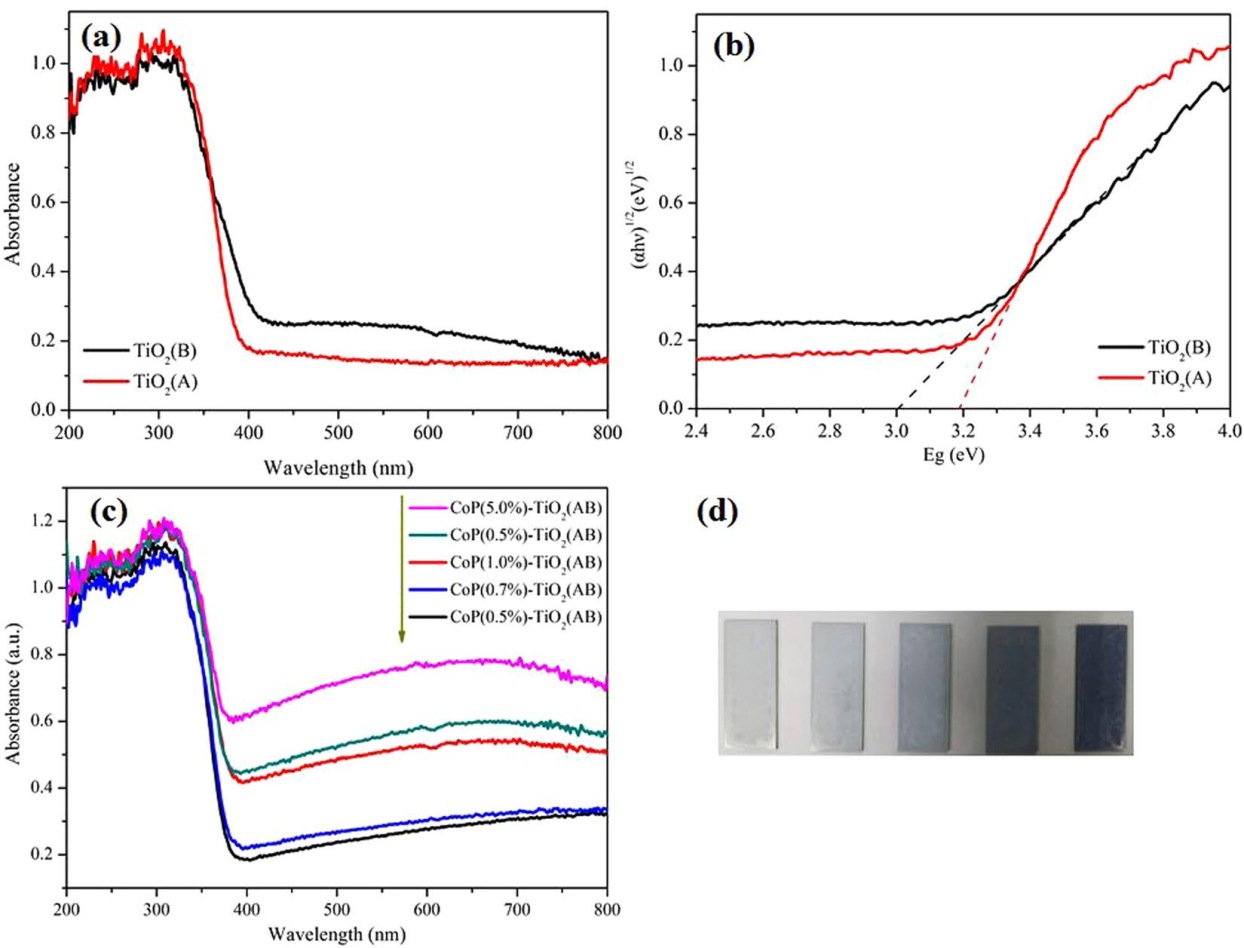
(**a**) UV-Vis DRS and (**b**) The plots of between (αhυ)^1/2^ and Eg for TiO_2_(A) and TiO_2_(B); (**c**) UV-Vis DRS of the CoP-TiO_2_ composites; (**d**) The optical photograph of the composite samples with different CoP content.

The optical properties of the TiO_2_(B) and TiO_2_(A) are investigated by UV-Vis diffuse reflectance spectra (DRS) as given in Fig. 4a. The absorption edges of pure TiO_2_(B) and TiO_2_(A) are 406 nm and 388 nm, respectively. According to Tauc formula [αhν = A(hν − Eg)^n/2^], the band gaps of TiO_2_(B) and TiO_2_(A) are estimated to be 3.05 and 3.19 eV, respectively (Fig. 4b). With the increasing loading of CoP, a red-shift DRS of is observed in the CoP-TiO_2_(AB) composite materials, which can be attributed to the absorption of CoP nanoparticles (Fig. 4c). However, there is no significant absorption edge change of the CoP-TiO_2_(AB) samples, indicating that CoP is incorporated onto the surface of TiO_2_(AB) rather than into the lattice^34^. Besides, the color of the composite photocatalyst becomes brown due to the absorption of CoP particles. The XPS spectrum results of TiO_2_(B), TiO_2_(AB), TiO_2_(A) and CoP are presented in Figure S4. The high resolution XPS spectrum shows O1s, Ti2p, P2p and Co2p, respectively. The binding energy of as-fabricated TiO_2_(B), TiO_2_(AB) and TiO_2_(A) is not changed for both Ti2p and O1s. In addition, the binding energy peaks of P 2p and Co (2p3/2) are in agreement with the reported ones^33^. Compared with TiO_2_(AB) (35.4 m^2^ g^−1^) sample, the as-prepared CoP(1%)-TiO_2_(AB) composite sample shows a small specific surface area of 30.53 m^2^ g^−1^ (Figure S5).

**Figure 5:**
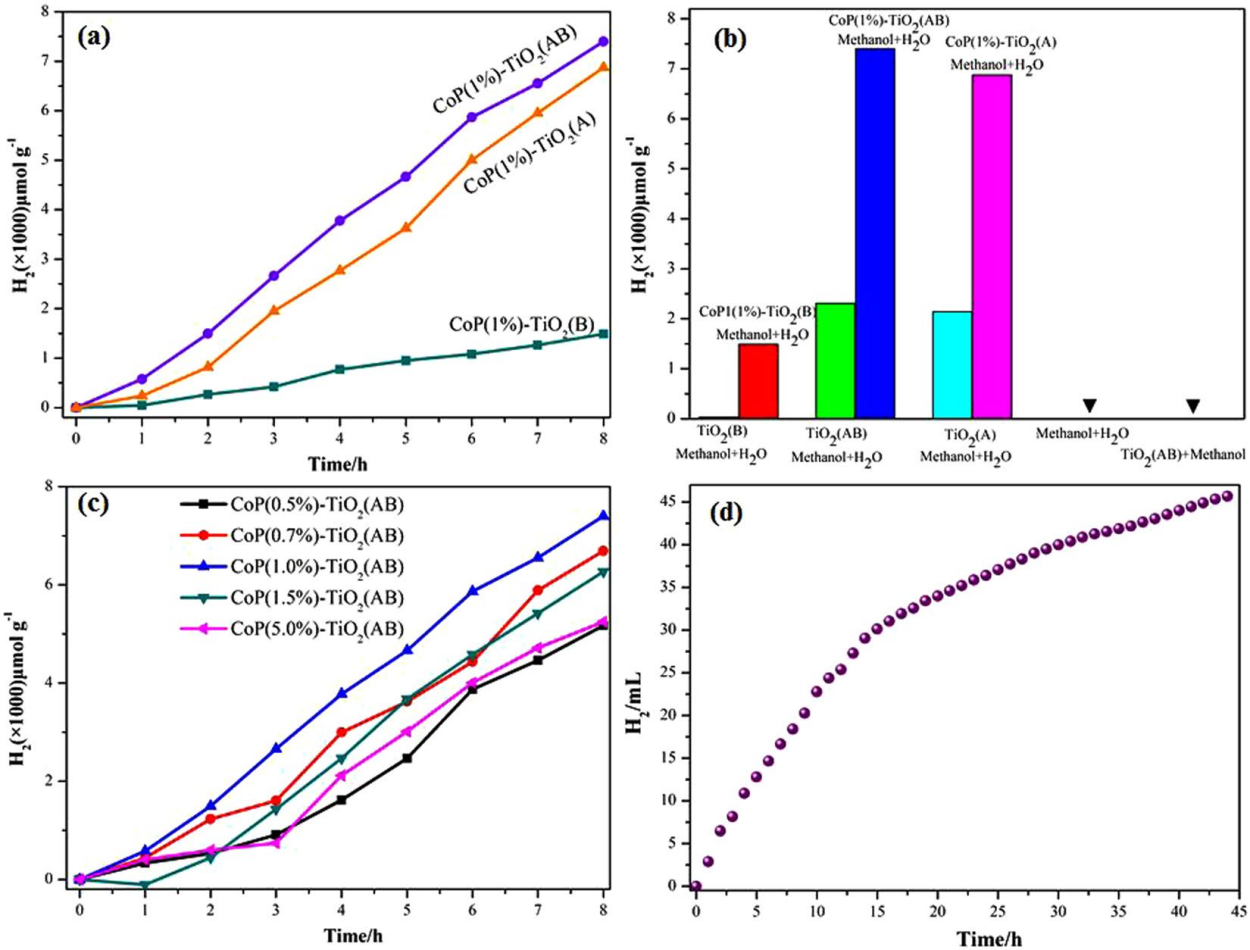
(**a**) The H_2_ generation rates of TiO_2_(A), TiO_2_(B) and TiO_2_(AB) with 1%CoP cocatalyst; (**b**) Activity comparisons between different TiO_2_ phase and CoP/TiO_2_ photocatalysts (**c**) Photocatalytic activities of CoP/TiO_2_(AB) hybrid photocatalysts with different CoP contents; (**d**) Photocatalytic durability test for CoP/TiO_2_(AB).

The photocatalytic performances of the as-prepared TiO_2_(A), TiO_2_(B) and TiO_2_(AB) samples are monitored for hydrogen production evolution from pure water containing methanol as sacrificial reagents under simulated sunlight irradiation. As plotted in Fig. 5a, it clearly seen that the core-shell structure TiO_2_(AB) catalysts express the highest yield of H_2_ with loading of 1% CoP cocatalysts. In the absence of cocatalysts, a sharp decrease of H_2_ production is observed for TiO_2_(A), TiO_2_(B) and TiO_2_(AB) samples. The highest H_2_-production rate of CoP(1%)- TiO_2_(AB) can be up to 7400 μmol g^−1^, which is about 3.2-times higher than that of TiO_2_(AB) (2306 μmol g^−1^) (Fig. 5b). The notably performance imply that CoP is an effective cocatalyst, which show impact on enhancing the photocatalytic activity positive. To check whether H_2_ produced is from water or methanol, blank experiments without catalysts is conducted. There is no H_2_ generation that suggested water is probably the real substrate in the photocatalytic reaction, and the conclusion is also verified by other authors^33,39^. The photocatalytic activity of the core-shell structure TiO_2_(AB) photocatalysts with different mount of CoP cocatalysts are also studied (Fig. 5c). As the amount of CoP increases, the H_2_ evolution rate is further enhanced, reaching the maximum value for CoP(1%)-TiO_2_. However, there is a decrease in the photocatalytic H_2_ evolution with further increase the loading amount of CoP particles. Hence, the optimal loading of CoP should be 1 wt%. The results may due to the following causes: (i) with the loading dosage of CoP cocatalysts, more active sites can be provided for reduction reactions; (ii) excessive CoP nanoparticles covered on the surface TiO_2_(AB) or aggregated into larger nanoparticles shielding surface active sites, which may lead to a negative impact on their catalytic activity. This phenomenon is similar to other nanoparticle cocatalysts^40–42^. Figure 5d displays the durability of the photocatalysts after 50 hours irradiation, and the result indicate that a slight increase is measured with CoP cocatalyst. XRD patterns further demonstrate the stable of the as-prepared photocatalyst (Figure S6).

**Figure 6:**
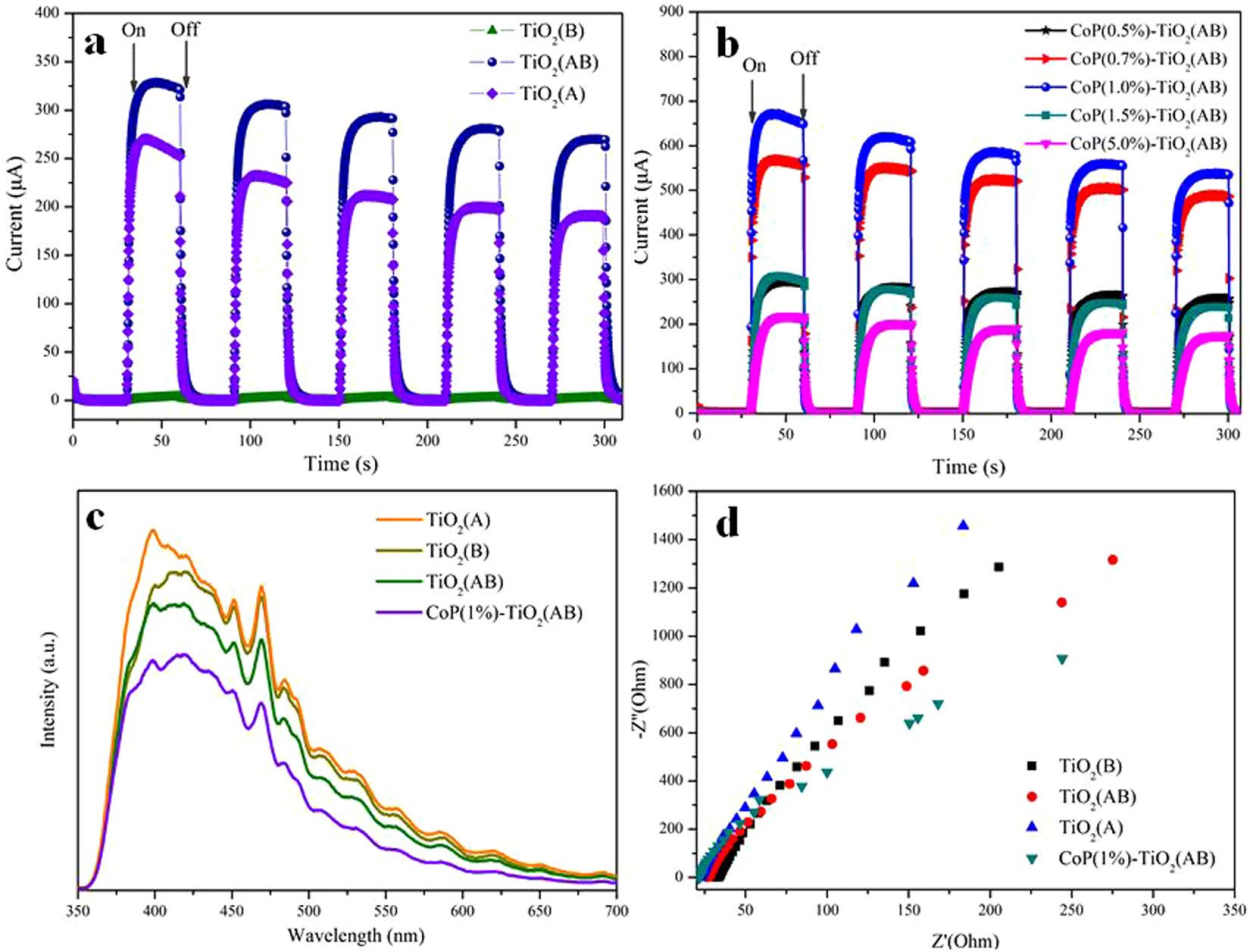
(**a**) Photogenerated currents density of the CoP(x%)-TiO_2_ photocatalysts and (**b**) different TiO_2_ phase under UV-visible light; (**c**) Photoluminescence (PL) spectra and (**d**) Nyquist impedance plots of TiO_2_(A), TiO_2_(B), TiO_2_(AB) and CoP(1%)-TiO_2_(AB) samples.

To further understand the enhancement of photocatalytic activity between Cop nanoparticles and TiO_2_(AB) photocatalysts, transient photocurrent response and electrochemical impedance spectroscopy (EIS) are conducted under UV-visible light irradiation. Upon illumination, the photocurrent immediately rises and the photocurrent rapidly decreases to zero as long as the light is switched off. As presented in Fig. 6a, the pulsed photocurrent density of the CoP(1%)-TiO_2_(AB) sample is higher than other photocatalysts, suggesting more efficient separation of photoexcited electron-hole pairs happened. In addition, CoP cocatalysts exhibit improved photocurrents (650 μA) compared with pure TiO_2_(AB) (Fig. 6b). It is notable that the change tendency agrees well with the photocatalytic H_2_ production activity. From the photoluminescence (PL) spectra in Fig. 6c, it can be seen that the emission intensity of the samples decreases in the following order: TiO_2_(A) < TiO_2_(B) < TiO_2_(AB) < CoP(1%)-TiO_2_(AB), indicating that charge recombination can be better suppressed after loading CoP cocatalysts. The PL emission bands at around 410 nm are observed in all samples, which belong to the emission of the band gap transition of TiO_2_^43^. Furthermore, the impedance radius of CoP(1%)-TiO_2_(AB) is smaller than TiO_2_(AB) (Fig. 6d), indicating that CoP can enhance separation efficiency of the photogenerated charges and facilitate the interfacial charge transfer.

## Mechanism of CoP-TiO_2_(AB)

**Figure 7:**
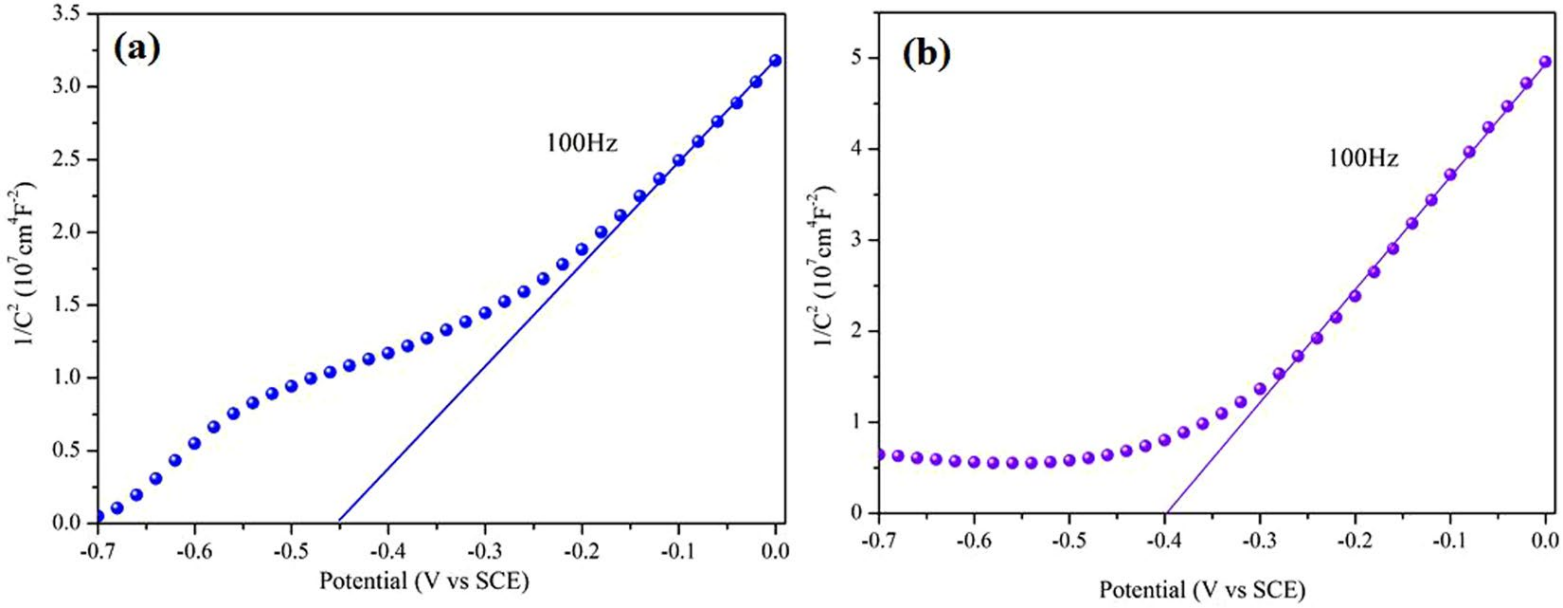
Mott-Schottky plot for (**a**) TiO_2_(A) and (**b**) TiO_2_(B) electrode in saturated Na_2_SO_4_ electrolyte solution (0.1 M, pH = 6.8) vs SCE.

**Figure 8:**
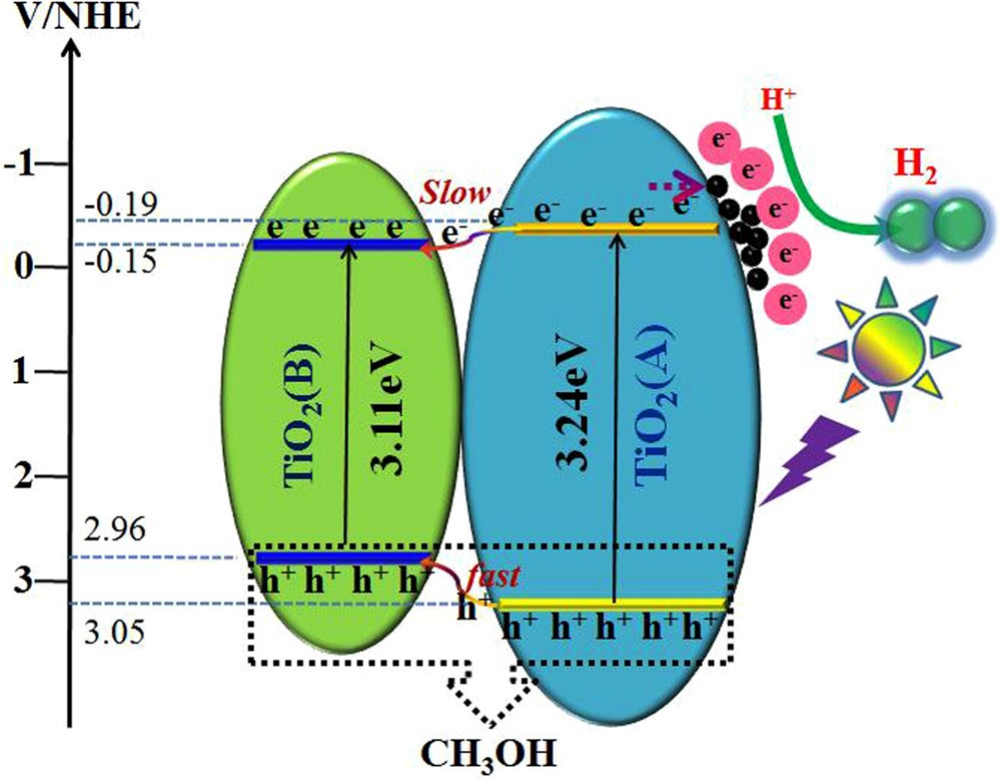
Proposed electron transfer and photocatalytic activity enhanced mechanism of CoP/TiO_2_(AB) upon UV-visible light irradiation.

In order to investigate the transfer mechanism of electrons (e^−^) and holes (h^+^) between TiO_2_(A) and TiO_2_(B), the band edge positions of conduction band (CB) and valence band (VB) of the two semiconductor are measured by Mott-Schottky measurement, which can directly probe the energy positions of TiO_2_(A) and TiO_2_(B). From Fig. 7, the CB and VB edges of TiO_2_(A) nanobelts are found to be −0.19 eV and +3.05 eV (vs. NHE), and the CB and VB edges of TiO_2_(B) nanosheets are −0.15 eV and +2.96 eV (vs. NHE), respectively. The calculation will be essential for the verification as shown in Support Information (Table 1) and early reports^35,43,44^. Accordingly, schematic diagram of the role of cocatalysts played in the separation and transfer of photogenerated charges of TiO_2_(AB) core-shell homojunction is shown in Fig. 8. Based on the energy structure of the interface between TiO_2_(A) and TiO_2_(B), the photoinduced holes of TiO_2_(A) could directly be injected into the VB of TiO_2_(B). Meanwhile, the excited electrons in TiO_2_(A) could be transferred to the CB of TiO_2_(B) easily under the irradiation of UV light. In the transfer process, the early literature has verified that the photogenerated holes can migrate more promptly to the adjacent TiO_2_(B) phase than the photogenerated electrons. Because the electrons migrate to the same destination in the same anatase crystals are 40 times longer (>40–160 ps) than those for holes (1–4 ps), which result in an accumulation of holes in the TiO_2_(B)^35^. These photoinduced holes at the VB of TiO_2_(A) and TiO_2_(B) can react with the H_2_O to generate some oxidation groups such as hydroxyl radicals, causing the oxidation reaction with the sacrificial reagent employed such as CH_3_OH. The overall outcome of the interphase charge migration is that a higher electron concentration left within the anatase shell. With the help of CoP cocatalyst, the photogenerated electrons on the CB of TiO_2_(A) tend to transfer to the loaded CoP nanoparticles, which are capable of the reduction reaction. As a consequence, the loaded cocatalysts are very helpful to the separation and transfer of photogenerated electron-hole pairs. Hence, the coherent interface between anatase and TiO_2_(B) structure and the CoP cocatalyst in the photocatalysis process may reduce recombination of photogenerated electron-hole pairs in the photocatalytic reaction and the photocatalytic activity is enhanced.

## Conclusion

In summary, a highly efficient core-shell TiO_2_(B)/anatase photocatalyst has been fabricated successfully for photocatalytic H_2_ evolution. The designed homojunction photocatalysts exhibited excellent performance compared with the pure phase, and a drastically enhanced photocatalytic activity appears with the participation of CoP cocatalysts. The H_2_-production rate of CoP(1%)-TiO_2_(AB) photocatalysts can be up to 7400 μmol·g^−1^. The remarkable activity is attributed to the cooperative contribution of effective core/shell structure that leading to the separation of photogenerated charges and the function of CoP cocatalysts on the transfer of photogenerated electrons from anatase to outside driven by the cocatalysts. This work demonstrated the great feasibility of utilizing CoP cocatalyst of different core/shell system in the application of photocatalytic H_2_- production.

## Electronic supplementary material

Supplementary Information
