# Supplementary material for: Highly Performance Core-Shell TiO2(B)/anatase Homojunction Nanobelts with Active Cobalt phosphide Cocatalyst for Hydrogen Production
Source: Sci Rep. 2017 Nov 6;7:14594. doi: 10.1038/s41598-017-15134-w (PMC5674065; doi:10.1038/s41598-017-15134-w)
Supplement: Supplementary file 1 — Supplementary Information [file 41598_2017_15134_MOESM1_ESM.pdf]

Supplementary information for

Highly Performance Core-Shell TiO_2(B)/anatase Homojunction Nanobelts with Active Cobalt phosphide Cocatalyst for Hydrogen Production

Guang Yang, Hao Ding*, Jiejie Feng, Qiang Hao, Sijia Sun, Weihua Ao, Daimei Chen*

Beijing Key Laboratory of Materials Utilization of Nonmetallic Minerals and Solid Wastes, National Laboratory of Mineral Materials, School of Materials Science and Technology, China University of Geosciences, Xueyuan Road, Haidian District, Beijing, 100083, P.R. China

* Corresponding Author E-mail: dinghao113@126.com
* Corresponding Author E-mail: chendaimei@cugb.edu.cn

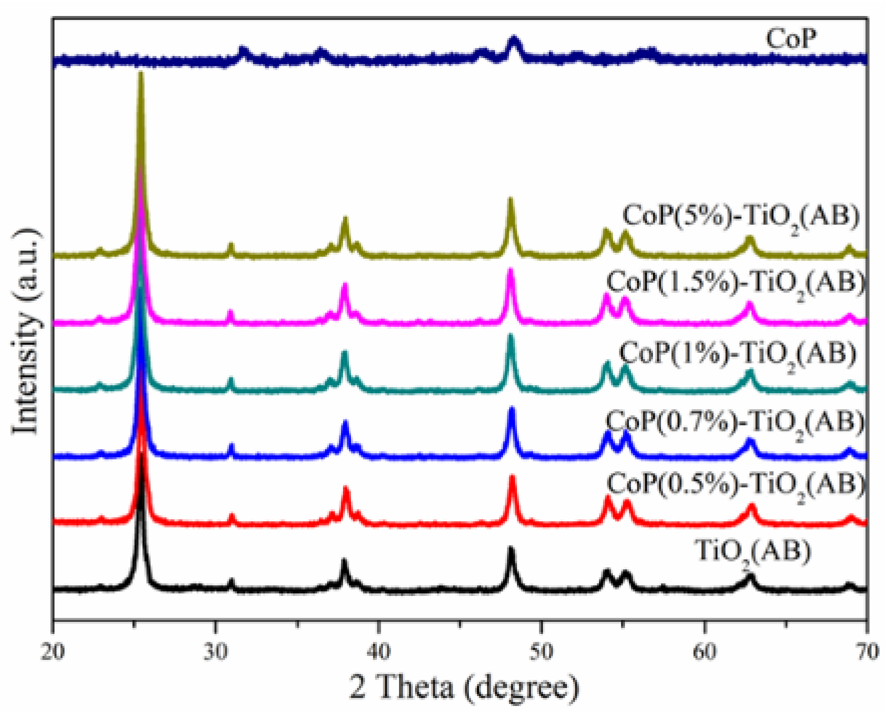

Figure S1 XRD patterns of the TiO_2(AB) photocatalysts with different amount of CoP nanoparticles

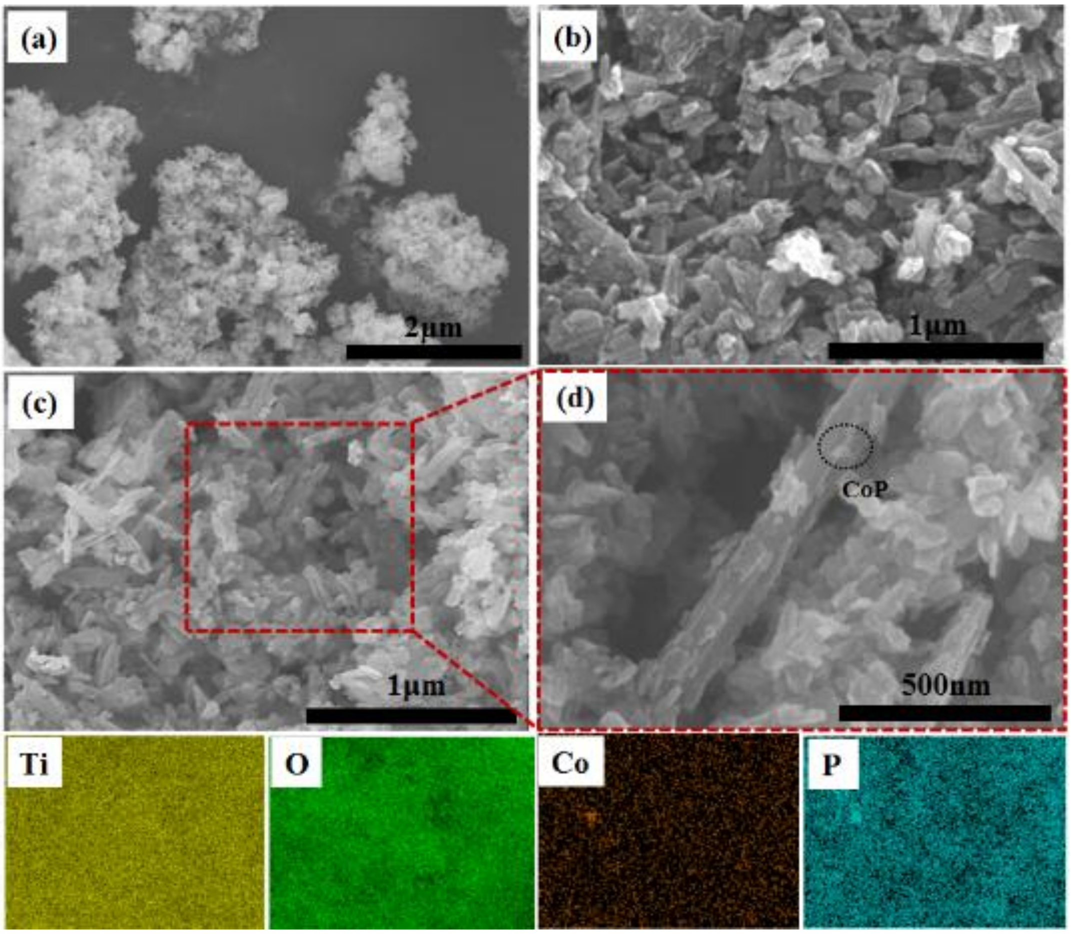

Figure S2 SEM of (a) CoP nanoparticles; (b) TiO_2(AB) core-shell structure; (c-d) CoP(1%)-TiO_2(AB) composite samples and the corresponding EDX elemental mapping of Co, P, Ti, and O of the CoP(1%)-TiO_2(AB) composite materials.

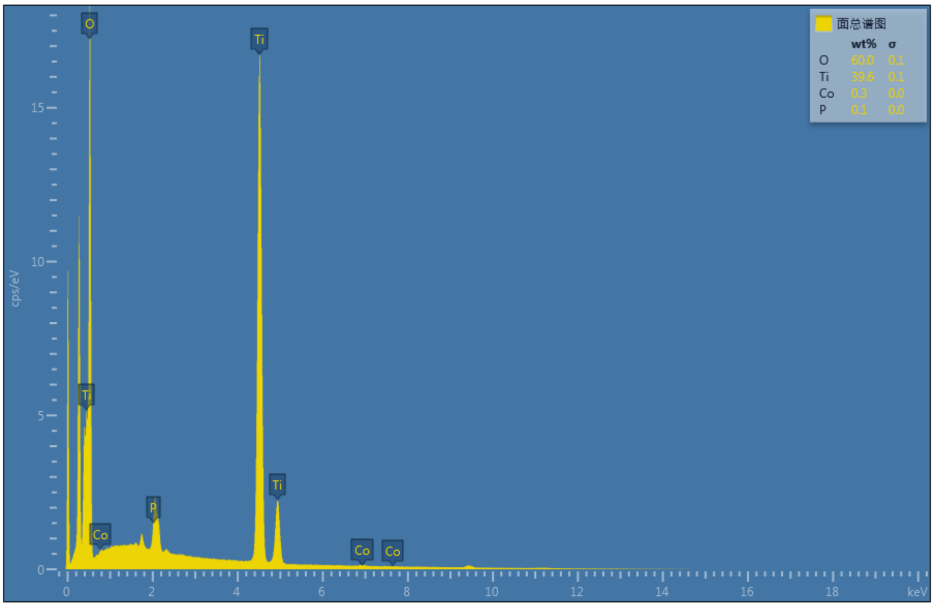

Figure S3 EDS patterns of CoP(1%)-TiO_2(AB) sample

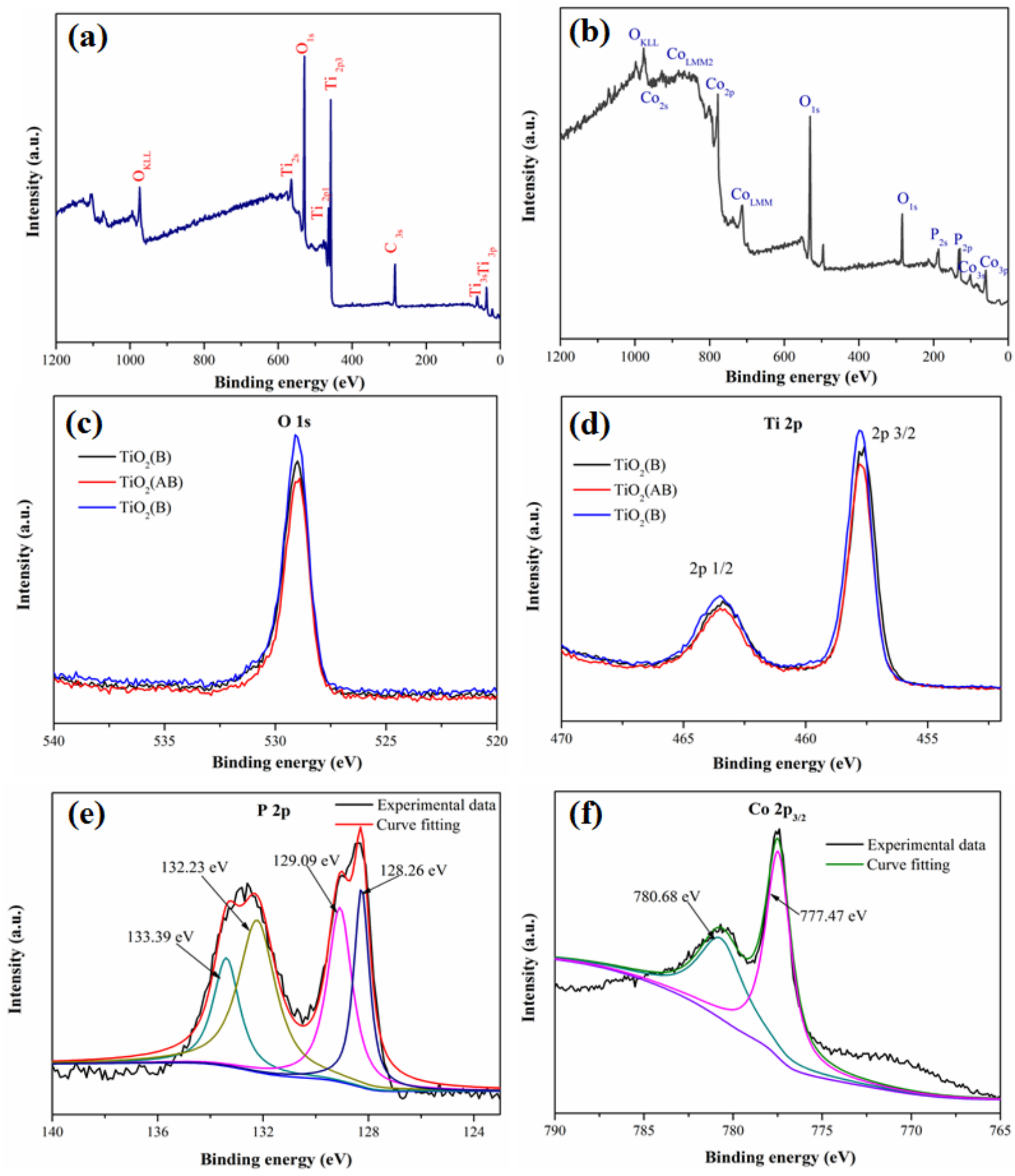

Figure S4 XPS spectra of (a) TiO_2(AB) and (b) CoP nanoparticles; High resolution XPS spectra of (c-d) TiO_2(B), TiO_2(AB) ,TiO_2(A) and (e-f) CoP nanoparticles

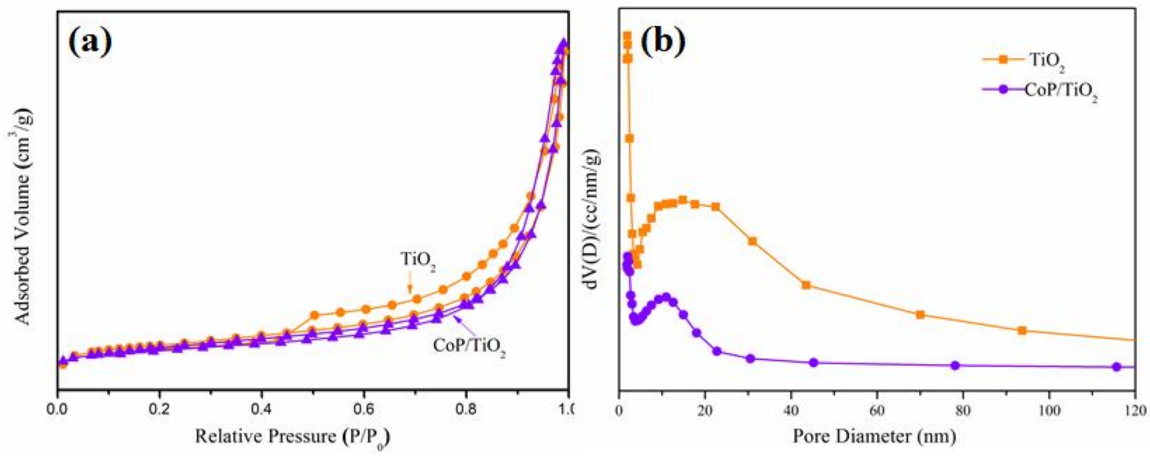

Figure S5 (a) N_2 adsorption-desorption isotherm and (b) pore size distribution curve of TiO_2(AB) and CoP(1%)-TiO_2.

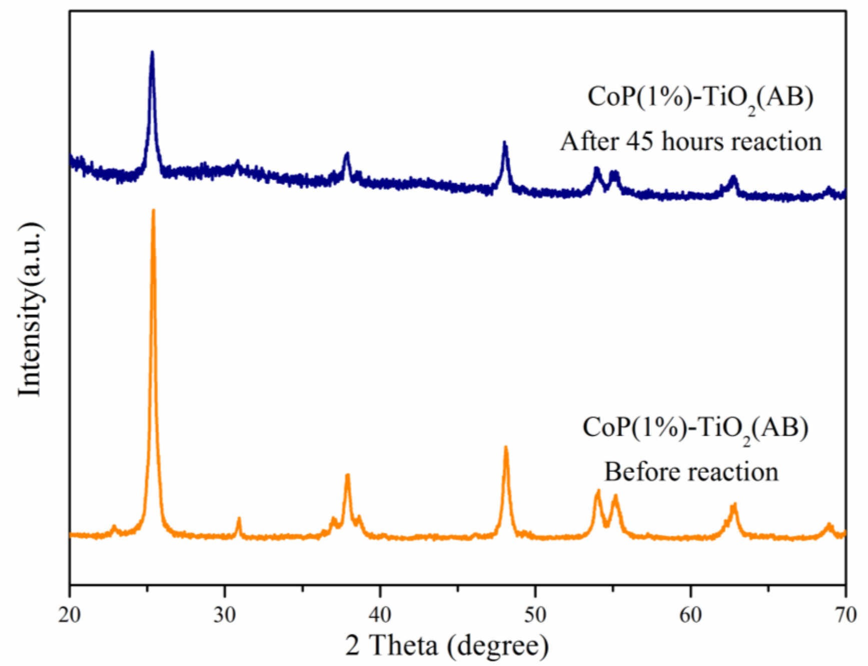

Figure S6 XRD patterns comparison of CoP(1%)-TiO_2(AB) sample before and after 45hours reaction

Table S1 Calculated band-gap energy (Eg), valence band edge potentials (E_{VB}), and conduction band edge potentials (E_{CB}) of the as-prepared TiO_2(A) and TiO_2(B) products by Mott-Schottky method

Semiconductor	Eg	Mott-Schottky method	
		E_{VB} (vs NHE)	E_{CB} (vs NHE)
TiO_2(A)	3.26 eV	3.14 eV	-0.12 eV
TiO_2(B)	2.65 eV	2.0 eV	-0.65 eV

Mott−Schottky equation:

$$\frac{1}{C^2} = \frac{2}{eN_d \varepsilon_0 \varepsilon}\left(V - V_{fb} - \frac{kT}{e}\right)$$

where C is the space charge capacitance, Nd is the donor density, ε and ε_0 are the dielectric constants of free space and the film electrode, respectively, V is the applied potential, Vfb is the flat-band potential, k is Boltzmann's constant, T is the temperature, and e is the electronic charge. The V_{fb} value can be determined from the extrapolation to $1/C^2 = 0$. The flat potential of TiO_2 nanobelt is calculated to be -0.43V versus the saturated calomel electrode (SCE), which is equivalent to -0.17 V versus the normal hydrogen electrode (NHE). It is known that the conduction bands of n-type semiconductors are 0-0.1 eV higher than the flat potentials, depending on the electron effective mass and carrier concentration [1, 2]. Here, the voltage difference between the conduction band and the flat potential is set to be 0.05 eV, and the bottom of the conduction band of TiO_2 is therefore -0.12 eV and the conduction band of $ZnIn_2S_4$ is 0.65 eV.
